# Modeling ALS with Patient-Derived iPSCs: Recent Advances and Future Potentials

**DOI:** 10.3390/brainsci15020134

**Published:** 2025-01-30

**Authors:** Ladan Dawoody Nejad, Erik P. Pioro

**Affiliations:** Djavad Mowafaghian Centre for Brain Health, Division of Neurology, Department of Medicine, University of British Columbia, Vancouver, BC V6T 1Z3, Canada; ladan.dawoodynejad@ubc.ca

**Keywords:** amyotrophic lateral sclerosis, astrocyte, co-culture, induced pluripotent stem cells, microglia, motor neuron, organoid, preclinical trials

## Abstract

Amyotrophic lateral sclerosis (ALS) is a terminal complex neurodegenerative disease, with 10–15% of cases being familial and the majority being sporadic with no known cause. There are no animal models for the 85–90% of sporadic ALS cases. More creative, sophisticated models of ALS disease are required to unravel the mysteries of this complicated disease. While ALS patients urgently require new medications and treatments, suitable preclinical *in vitro* models for drug screening are lacking. Therefore, human-derived induced pluripotent stem cell (hiPSC) technology offers the opportunity to model diverse and unreachable cell types in a culture dish. In this review, we focus on recent hiPSC-derived ALS neuronal and non-neuronal models to examine the research progress of current ALS 2D monocultures, co-cultures, and more complex 3D-model organoids. Despite the challenges inherent to hiPSC-based models, their application to preclinical drug studies is enormous.

## 1. Introduction

Amyotrophic lateral sclerosis (ALS) is characterized by the degeneration of upper and lower motor neurons (LMNs) in the motor cortex, brainstem, and spinal cord, resulting in the progressive weakness of voluntary and respiratory muscles. The disease’s rapid progression limits median survival to between 2 and 5 years, with no effective treatment available [1,2]. Between 85% and 90% of ALS cases are sporadic (sALS), with unknown causes that most likely combine environmental and polygenic factors [3]. Therefore, developing sALS disease models is very challenging. An estimated 10–15% of cases are familial ALS (fALS), which are linked to monogenic causes with primarily dominant inheritance [1,4]. ALS is now recognized as a multisystem neurodegenerative illness with heterogeneity in its clinical, genetic, and neuropathological aspects [1]. In fact, the recognized overlap with frontotemporal dementia (FTD) suggests ALS and FTD are now thought to be two ends of a spectrum due to the overlap in molecular mechanisms underlying both neurodegenerative disorders [5].

**Figure 1:**
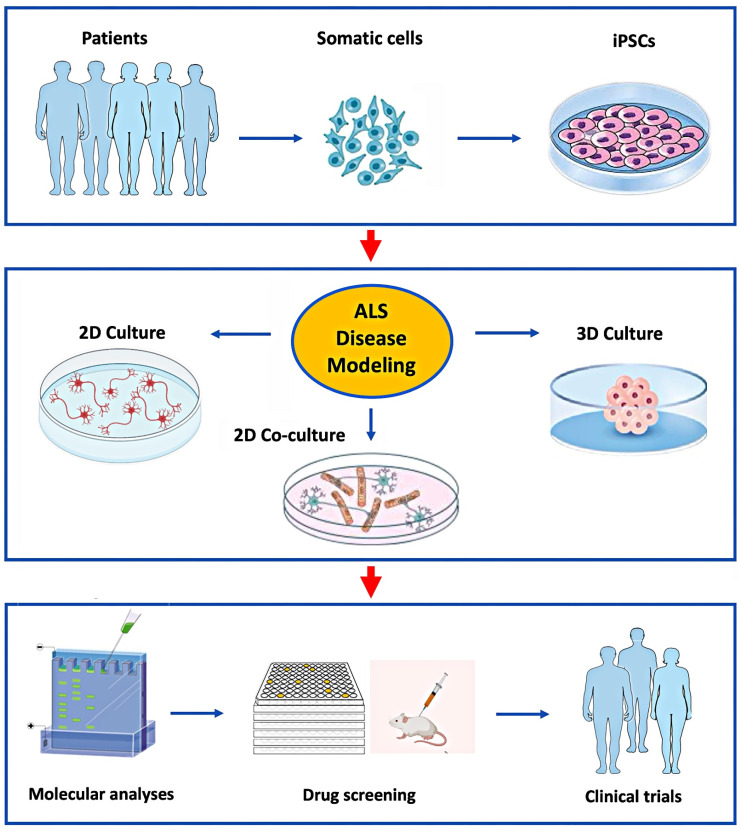
Application of hiPSC’s to model ALS *in vitro* for studies of disease mechanisms and drug development.

In 2007, Takahashi et al. demonstrated how to reprogram somatic cells into induced pluripotent stem cells (iPSCs), allowing researchers to differentiate these cells and model human disease processes [6]. Since then, iPSC biology has progressed rapidly, with the discovery of more advanced reprogramming techniques [7,8]. These developments have enabled *in vitro* investigations with human (h) iPSCs into the mechanisms of ALS, the most common adult motor neuron disease (MND) [9]. hiPSCs can differentiate into any somatic cell type found in the central and peripheral nervous systems (CNS and PNS), including neurons, astrocytes, oligodendrocytes, microglia, Schwann cells, and myocytes. The most notable feature of hiPSCs is the ability to be generated from somatic cells (usually skin fibroblasts or peripheral blood mononuclear cells) during adulthood (Figure 1). This enables the creation of iPSCs from ALS patients with varied phenotypes and genotypes, providing insights into the range of both familial and more common sporadic cases [10]. Recent whole-genome sequencing studies have shown that sporadic ALS cases are linked to several variants, suggesting that accumulating genetic mutations cause the disease to manifest [11,12]. The hiPSC containing all of the donor’s genetic material can be differentiated into a variety of cell types to be a powerful tool for simulating diverse sporadic disease pathogenic mechanisms *in vitro*. Importantly, hiPSC-based cellular models and their pathologies are driven by genetic information even in sporadic cases, because most epigenetic modifications in donor cells are reset to the embryonic state during the iPSC reprogramming process, with the exception of genetic imprinting and epigenetic memory [11].

Patient-derived somatic cells can be reprogrammed to iPSCs (top panel) and then differentiated into various cell types grown in 2D or 3D culture systems (middle panel). This allows for a detailed study of the molecular mechanisms causing ALS in specific patients for the development and preclinical testing of novel drugs selected for clinical trials (lower panel).

The more recent stem cell technologies, particularly those using hiPSCs, have enormous potential for modeling the distinct pathophysiology of an individual patient. Nevertheless, certain limitations exist, including phenotypic and genotypic variability and inadequate maturation, in comparison to *in vivo* models. In addition, the majority of preclinical ALS research to date has been conducted *in vivo* using mouse models [13,14]. Animal studies, however, are less than ideal for large-scale drug testing due to their high degree of variability and ethical issues [14], among other reasons. Although *in vitro* tissue culture models are popular methods for studying ALS pathogenic mechanisms and finding novel treatments because of their simplicity and lower cost, they cannot fully recapitulate human ALS. However, advancements with intricate *in vitro* cultures and co-cultures using organoids and microfluidic technology have made it possible to create large model systems for a more representative study of ALS.

No cure or effective treatment is currently available for ALS due to its great mechanistic complexity, genetic variability, and translational gaps between animal models and this human disease. The absence of proper preclinical *in vitro* models poses a hurdle to suitable clinical studies in ALS. Several recent reviews have covered different aspects of the hiPSC-derived *in vitro* modeling of ALS [10,15,16,17,18,19,20,21]. In this review, we discuss some of the most recent complex hiPSC-derived ALS models, such as monoculture differentiation, two-dimensional (2D) co-culture compositions, and three-dimensional (3D) organoids and their applications to understanding disease mechanisms and recent preclinical drug testing.

## 2. Human iPSC-Derived Motor Neuron Culturing

A variety of animal organisms, including yeast [22], worms [23], fruit flies [24], zebrafish [25], and rodents [26], have been applied to model ALS. hiPSCs have enormous potential in this effort, because they can be differentiated into multiple cell lineages, including motor neurons (MNs), while maintaining the patient’s genetic characteristics [11]. Such hiPSC technology can improve disease modeling, elucidate how drugs work, and possibly design new therapies. *In vitro* models are ideal for high-throughput screening and are advantageous in controlled, low-cost experimental settings. Even though there are many benefits to hiPSC disease modeling, several challenges exist, including obtaining disease-specific cell lines, preserving and differentiating cell cultures, and guaranteeing the models’ resilience and repeatability.

Since most ALS patients do not have a family history of the disease, modeling sALS is crucial to developing novel therapies. However, the absence of known monogenic mutations supports the notion of polygenic influences, along with environmental factors, in the causation of sALS. In a recent study, researchers at Keio University generated iPSCs from 32 sALS patients and six healthy individuals and established various *in vitro* cellular models as sALS resources [11]. They then differentiated these cells into MNs, which maintained the patient’s inherent genetic information and observed variations in the onset and development of various abnormalities, including the patterns of neuronal degeneration, aberrant protein aggregation, and mechanisms of cell death pathways. Finally, they created a case-clustering method to categorize these heterogeneous sALS models according to their *in vitro* characteristics. This study used multiple-patient sALS models to first categorize genetically and clinically heterogeneous sALS patients and then used a multiphenotypic analysis/screening system to identify potential drug candidates targeting pathogenic mechanisms in both sALS and fALS. As a result, ropinirole hydrochloride (ROPI), a dopamine D2 receptor (D2R) agonist, was identified as a promising drug candidate. Although ROPI showed protective effects in FUS- and TDP-43-mutated fALS models and the majority of sALS models, it did not suppress detected phenotypes in SOD1-mutant ALS models [11]. Whether the drug’s neuroprotective effect is mediated via a D2R-dependent or -independent mechanism, or both, is unclear, as this receptor is present on human spinal cord MNs and is expressed by ALS patient iPSC-derived spinal MNs. This finding raises the possibility that ROPI may protect cells from mitochondrial dysfunction occurring in ALS [27,28]. This study demonstrated ROPI’s neuroprotective effect on MNs derived from hiPSCs obtained from 9 out of 22 sALS patients as well as from patients carrying FUS (*n* = 9) and TDP-43 (*n* = 4) mutations compared to three healthy controls [11]. Lack of the drug’s benefit on cells from the six sALS patients supports the notion that pathogenic mechanisms, and therefore response to therapies, likely differ between individuals with sALS. Based on this hiPSC-related *in vitro* drug-discovery study, a phase 1/2a trial of ROPI was performed in patients with sALS [27]. Details of the clinical trial findings are beyond the scope of this hiPSC-related review, but several measures revealed a protective effect against ALS, including a slowed decline of ALSFRS-R scores and almost 28 weeks of disease-progression-free survival during the open-label extension period as well as a reduction in CSF neurofilament and lipid peroxide levels [27]. ROPI protected FUS-ALS, TDP-43 ALS, and sALS hiPSC-derived MNs by preventing reactive oxygen species formation, neurite degeneration (preserved length), neurotoxicity (reduced LDH leakage), apoptosis (decreased cleaved caspase-3), and TDP-43 aggregate formation (except in FUS-ALS MNs where FUS aggregates were prevented) [11]. Interestingly, ROPI showed no similar benefits on SOD1-positive iPSC-derived MNs except for improved mitochondrial dysfunction, suggesting the latter abnormality is common to both non-SOD1-positive and SOD1-positive forms of ALS. Consequently, these findings demonstrate the potential benefit of ROPI as an ALS treatment for a variety of clinical scenarios. The study also discovered that in most sALS models, lipid peroxidation and resultant ferroptosis are critical factors in the degeneration of MNs. Overall, the study highlights the utility of iPSC technology to provide new insights into pathogenic mechanisms underlying sALS and novel avenues for identifying effective treatments [11].

Studying iPSC-derived MNs and related cells from patients with genetic and sporadic forms of ALS will help clarify its causative molecular mechanisms and provide a model for drug discovery. It may be possible to model genetic variants of ALS with fewer patient lines if isogenic lines can be produced as controls. Nonetheless, modeling sALS with varied etiologies becomes more complicated [29]. Although in the previous Japanese study, only 32 ALS patients and six controls were adequate to identify distinct differences between groups of sALS patients [11], hiPSC biology is likely to vary among different ethnicities. Differences in the distributions of genes causing fALS in various countries support this notion. For example, the *C9orf72* mutation is the most common genetic cause of fALS in Caucasians (~40%) but is much less frequent in Asian populations. In a study of 563 Japanese ALS patients, only 0.4% of sALS and no fALS cases were linked to *C9orf72* [30]. Conversely, *SOD1* mutations are more prevalent in Asian cohorts compared to Western ones, at 27.9% of 86 Chinese fALS cases [31]. These variations highlight the importance of considering genetic diversity in ALS research and treatment approaches across different ethnic groups. Therefore, studies on iPSCs from more patients may be required to identify additional and personalized disease-specific processes.

Answer ALS, a comprehensive research initiative aimed at understanding and finding a cure for this disease, is developing a large-scale hiPSC repository of at least 1000 iPSC lines generated with a standardized protocol from both ALS patients and healthy controls, along with clinical and whole-genome sequencing information [29]. Four hundred and thirty-three hiPSC lines were used in a study to differentiate into MNs via a specific 32-day protocol with a subsequent examination of gene expression patterns and neural cell markers. Patient covariates were considered to identify the confounding variables and challenges in these extensive patient-derived iPSC differentiation studies, providing valuable resources to the community [29]. Characterizing this largest batch of hiPSCs, which were differentiated into MNs, followed by bulk RNA-seq and cell marker expression profiling, revealed that sex and cell composition are significant sources of heterogeneity that must be carefully controlled in future research. Interestingly, Islet-1 (ISL1)-positive MN staining was more common in ALS cases than in controls. Additionally, the sex of the hiPSCs separated the data into two distinct subgroups independent of disease status. Compared to females, male cells produced more MNs with several dysregulated genes that were enriched in stress-related pathways [29].

hiPSCs are traditionally programmed into MN lineages by adding growth hormones, pluripotency inhibitors, and other neuron patterning factors to the media [32]. An alternative method for a quicker neuronal differentiation of hiPSCs involves genetic reprogramming to express master regulators of MN development by reducing or removing intermediate progenitor stages [33]. According to a 2017 study, the development of MN identity in mouse embryonic stem cells (mESCs) is dependent on the expression of only three transcription factors called NIL factors: neurogenin 2 (NGN2), ISL1, and LIM homeobox proteins (LHX3) [33]. In order to initiate neural differentiation and survival programs in the CNS and PNS, NGN2 is produced in neuronal progenitor cells (NPCs); LHX3 and ISL1 form a heterodimer transcription regulator complex to activate genes driving postmitotic neuron specification [34,35]. Standard methods used to develop hiPSCs into MNs are time-consuming and often result in heterogeneous populations of neuronal subtypes. Thus, to achieve the therapeutic potential of hiPSCs, additional refinement is required. Several research groups have proposed the direct reprogramming of stem cells into MNs by inserting recombinant genes that encode such transcription master regulators [33,34,36]. In these studies, transgene cassettes are introduced into ESCs or hiPSCs via retroviral vectors. However, because transgenic integration into the recipient genome occurs randomly, the potential for clinical applications has raised safety concerns.

According to a 2023 study [35], scientists used CRISPR/Cas9 (clustered regularly interspersed short palindromic repeats/CRISPR-associated protein 9) gene editing techniques with high-fidelity recombinant SpCas9 to unidirectionally transform iPSCs into MNs. ISL1 and LHX3 transgenes were delivered into well-defined safe harbor locations (H11, ROSA26, and AAVS1) in the human iPSC genome by electroporation. This method significantly reduced off-target binding of the Cas9/gRNA complex, because it is transient and resulted in much lower levels of ribonucleoprotein complexes compared to methods using plasmid DNA transfection to encode Cas9 and sgRNA. It was discovered that transgene transcription factors become activated in the modified iPSCs even prior to adding a TRE-inducing agent (doxycycline). This established a genetic program dedicated to MNs’ fate, although it was unable to carry out the unidirectional transition to MNs in the absence of extrinsic neuron patterning factors provided in the differentiation media. A comparative global transcriptome analysis of MN formation in native and LHX3/ISL1-modified iPSC cultures revealed that the neuronal patterning process was facilitated by genetic factors. However, premature activation of genetic pathways typical of mature MNs was caused by leaky gene expression of the exogenous MN master regulators in iPSCs. In addition, dysregulation of metabolic and regulatory pathways within the developmental process altered MN electrophysiological responses. Premature activation of ISL1 and LHX3 transgenes in iPSCs impacted neuronal development, cell proliferation, and Ca^2+^ signaling pathways in mature MNs; it also changed gene expression patterns during all phases of differentiation. This indicates that early activation of MN genes interrupts the correct differentiation process. Overexpressing important transcription factors in hiPSCs with CRISPR/Cas9 technology prevents the expected unidirectional transition to MNs [35].

The *in vitro* generation of functional MNs involves exposing them to intricate formulations that include the following neuron patterning molecules: retinoic acid (RA), WNT/sonic hedgehog (SHH) pathway activators, and notch pathway inhibitors. These formulations are supplied at precisely defined concentrations and within specific time windows for effectiveness [32]. Phrenic MNs (phMNs) innervating the diaphragm almost always degenerate in ALS, which results in respiratory failure and death [37]. Our understanding of the mechanisms underlying phMN degeneration in ALS has been poor primarily because of limited human experimental models to investigate such MNs. phMNs differ from other spinal MNs in several aspects, including their developmental origin, unique architecture, and electrical characteristics. The most well-established and widely utilized hiPSC-based MN derivation techniques produce heterogeneous cultures composed of MNs from the lateral motor column (LMC) and median motor column (MMC). Because the absence of phMNs from these cultures prevents the study of their (patho) physiology, a technique using hiPSCs to produce phMN-enriched cultures in as little as 30 days has been developed. This highly reproducible technique employs multiple hiPSC lines, combines optimal concentrations of RA and the SHH agonist purmorphamin, and uses fluorescence-activated cell sorting (FACS) based on a cell-surface protein immunoglobulin superfamily DCC subclass member 3 (IGDCC3) to enrich for phMNs. The calibrated activation of RA and SHH signaling in hiPSC-derived neural progenitor cells (NPCs) facilitates a cervical identity of dorsal NPCs to produce phMN-like neurons. This novel methodology now allows for the study of disease-relevant cells to evaluate mechanisms of respiratory MN dysfunction in ALS [37].

Neurons form highly specialized functional units with neuroglia (i.e., astrocytes, oligodendrocytes, and microglia) with close interactions that influence their health and survival. For this reason, we will discuss recent neuronal–neuroglial co-culturing studies that aim to more closely model the degeneration occurring in the CNS of patients with ALS.

## 3. Co-Culturing hiPSC-Derived Motor Neurons with Neuroglia

In addition to MNs, other neuronal subtypes and neuroglia, including astrocytes, are affected by ALS. Approximately 20–40% of neuroglia in the CNS are astrocytes, making them one of the most prevalent glial cell types [38]. Their functions are multiple, including maintaining brain and spinal cord structural integrity, providing neurotrophic support and neurotransmitter control and modulating neural activity. By controlling the blood–brain barrier, water flux, and ion and pH homeostasis and eliminating reactive oxygen species, astrocytes help to maintain the ideal CNS environment. Additionally, they contribute to inflammatory and immunologic responses by secreting cytokines and phagocytozing cellular debris and helping to create boundaries after injury. Through a variety of mechanisms, including creating perisynaptic processes (PAPs) by their endfeet and secreting specific molecules such as thrombospondins (THBS1/2), glypicans (GPC4/6), transforming growth factor β1 (TGF-β1), and brain-derived neurotrophic factor (BDNF), astrocytes modulate the plasticity and function of neuronal synapses [38]. In the CNS, these chemicals facilitate the development and maturation of inhibitory synapses, excitatory synapses, and tumor necrosis factor-α (TNF-α). In addition, astrocytes and microglia work together to dynamically modulate neuroplasticity, which is crucial for memory and learning [38]. Recent human embryonic stem cell experiments co-culturing MNs with astrocytes expressing the mutant SOD1 enzyme reduce MN survival [39,40,41,42].

Microglia, another type of neuroglia in the brain and spinal cord, receive signals from astrocytes and are required to properly assemble complex neural networks [43]. They account for about 10–15% of all cells in the brain [44]. Although derived from blood-borne macrophages, microglia differ from other tissue macrophages due to their specific homeostatic nature and multiple roles, including controlling brain growth, maintaining neural networks, and repairing injuries [45]. They remove bacteria, dead cells, unnecessary synapses, aggregated proteins, and other soluble and particulate antigens that could harm the CNS. Microglia are also important mediators of neuroinflammation and have the ability to initiate or influence a wide range of cellular responses, as they are the main source of proinflammatory cytokines. Brain aging and neurodegeneration are associated with changes in microglial functioning [45]. Microglia have also been found to express significant levels of *SOD1* and *C9orf72*, two important ALS-associated genes [46]. There are several species-specific characteristics of microglia that are different between humans and rodents [47], with several genes linked to neurodegenerative diseases of microglia lacking orthologs in mice [48]. Furthermore, the precise function of microglia in the pathophysiology of ALS remains unclear, and, therefore, more realistic human disease models of microglial dysfunction are needed.

### 3.1. Astrocyte Co-Cultures

In the past, astroglia were thought of as simply passive satellite cells in the CNS, supporting neurons metabolically and controlling extracellular homeostasis. However, recent research has demonstrated their capacity to receive signals from neurons and release neuroactive chemicals as well as actively regulate neuronal functions, communication pathways, and plasticity [49].

Astrocytes have been implicated in contributing to MN toxicity and death in fALS by enhancing the release of toxic substances, decreasing lactate synthesis, and diminishing glutamate uptake [42]. FUS-ALS iPSC-derived astrocytes were studied in a monoculture and co-culture with FUS-ALS MNs in a microfluidic device with healthy skeletal myocytes to examine their interactions [50]. Compared to isogenic control astrocytes, FUS-ALS astrocytes were found by a variety of techniques, including immunocytochemistry, RNA sequencing, and calcium transient hyperactivity, to display elevated glial fibrillary acidic protein (GFAP) expression, cytoplasmic FUS mislocalization, the production of inflammatory cytokines, and enhanced spontaneous reactivity. Such co-cultures of hiPSC-derived cells mimicking *in vivo* multicellular networks revealed the deleterious effects of FUS astrocytes on MN neurite outgrowth and network integration, as well as on neuromuscular junction (NMJ) formation and functionality. Overall, FUS-ALS astrocytes, especially those expressing a mutation associated with an earlier onset and more aggressive clinical course, contributed to disease via multiple gain-of-toxicity and loss-of-support mechanisms. The increased secretion of proinflammatory cytokines by mutant-expressing astrocytes indicates that this is not dependent on stimulation by microglia [40], since the latter were missing from the cultures, and supports astrocytic contribution to neuroinflammation in ALS [51]. The study also found that activation of the WNT/β-catenin pathway plays a significant, although complex, role in FUS-ALS, since it may have some neuroprotective functions and is normally involved in the development of NMJs, axonal guidance, and cell survival [52]. The author’s multicellular microfluidics model provides a platform for more research with additional hiPSC lines to further explore the etiology of astrocyte cytotoxic characteristics and for novel drug development and testing.

ALS with frontotemporal dementia (ALS-FTD) is often caused by a polymorphic hexanucleotide repeat expansion (HRE) of GGGGCC (G4C2) in the first intron of the *C9orf72* gene from normally less than 30 repeats into the hundreds or thousands, pathologically. The unusual repeat-associated non-AUG (RAN) translation of the HRE into five toxic dipeptide repeat (DPR) species—poly-PA, poly-GA, poly-PR, poly-GR, and poly-GP—appears to be a key factor driving the pathophysiology of *C9orf72*-related ALS-FTD. The most hazardous of the five DPRs are believed to be those containing arginine, specifically, poly-GR and poly-PR [53,54]. It has been shown that these DPRs affect the development of membrane-less organelles such as stress granules and can result in DNA damage and mitochondrial malfunction. Their expression is toxic to mouse and hiPSC-derived cortical and MNs [55,56].

Co-culturing iPSC-derived human astrocytes (iAstrocytes) and Hb9-GFP mouse MNs investigated differences in the cell-to-cell propagation of *C9orf72*-related poly-GA oligomers and fibrils [55]. In this system, DPR species are promptly internalized by astrocytes and spread to neuronal units with fibrils transmitting to MNs six times more efficiently than oligomers. Poly-GA DPRs are internalized through both dynamin-dependent and -independent endocytosis, ultimately localizing in lysosomes and causing axonal swelling. These findings point to a significant involvement of astrocytes in the transfer of DPRs produced from mutated *C9orf72* to nearby MNs. It should be noted that results obtained in cell culture may not directly apply to humans or *in vivo* animal models. For example, the heterogeneous pattern of *C9orf72* expression endogenously *in vivo* may result in significant regional variations in DPR concentrations in patients. In addition, the short-length DPR used in this study may affect cellular functions differently from the longer DPR repetitions found in patients with *C9orf72* ALS. Nonetheless, this research sheds light on the basic principles of the biology behind poly-GA aggregation, binding, absorption, and cell-to-cell propagation between glia and neurons [55].

A number of investigations of the *C9orf72* HRE mutation in humans or animals have shown an increased tendency to develop autoimmune diseases [57,58]. These studies collectively imply that *C9orf72*-induced ALS could arise from either the direct or indirect dysregulation of one or more inflammatory pathways. Previous research has revealed that a pathological feature of ALS is neuroinflammation, which is already present in the early stages of the disease. According to autopsy data, activated microglia and astrocytes are primarily responsible for inducing such inflammatory changes [59]. Although neurons may not have major roles in the inflammatory process of ALS, changes in their cellular homeostasis increase their susceptibility to inflammation and subsequent neurotoxicity [60].

### 3.2. Microglia Co-Cultures

According to recent research, *in vitro* pluripotent stem cell-derived microglia-like cells have been generated from multiple disease-specific cell lines and accurately replicate the expected ontogeny and characteristics of their *in vivo* counterparts in organotypic 3D cultures [61]. A cell model to explore human MN–microglia crosstalk has been recently reported by using a co-culture system of iPSC-derived microglia and spinal MNs [46,62,63]. In a co-culture, such MNs exhibit appropriate cellular markers, including neuronal electrophysiology by whole-cell patch-clamp and calcium imaging, both spontaneously and in response to potassium chloride stimulation. In addition to expressing important ALS-associated genes, co-cultured microglia display direct interactions with MNs and their neurites, exhibit ramifications with extremely dynamic remodeling, and have a gene profile similar to primary human microglia. This model can be used to investigate both cell-autonomous and non-cell-autonomous phenotypes, is relatively simple and highly efficient, and allows for easy scaling up for large-scale and drug-screening experiments [46].

Widespread microglial activation has been linked to the progression of ALS, especially in patients with *C9orf72* HRE, the most prevalent genetic cause of the disease [64]. In neurons, the *C9orf72* HRE causes haploinsufficiency, the formation of RNA foci and DPRs, and cytoplasmic mislocalization of TDP-43. In an RNA sequencing analysis of hiPSC-derived *C9orf72* HRE mutant microglia, pathways associated with immune cell activation and the production of cytokine CXCL1 and CXCL6 activity were shown to be enriched, particularly after lipopolysaccharides (LPS) priming. LPS is one of several triggers that can activate microglia and cause a pro-inflammatory phenotype that increases neurotoxicity [64]. LPS-primed *C9orf72* HRE mutant microglia consistently upregulate matrix metalloproteinase-9 (MMP9) production and release. MMP9 is an endopeptidase that cleaves cell-surface receptors as well as a number of extracellular matrix components. Multiple neurodegenerative diseases share MMP9 dysregulation, including ALS, where MMP9 has been shown to have a neurotoxic effect [65,66]. Unstimulated *C9orf72* mutant microglia in an hiPSC-derived microglia–MN co-culture assay exhibit a dysregulated supernatant profile, although without impacting the survival or activity of normal/healthy spinal MNs. After LPS exposure and priming, however, *C9orf72* mutant microglia co-cultures cause obvious neurodegeneration and trigger apoptosis in healthy MNs. The application of an MMP9 inhibitor concurrently with LPS-stimulated *C9orf72* mutant microglia reduces their neurotoxic properties apparently mediated via the dipeptidyl peptidase-4 (DPP4) they release [64].

Although individual astrocyte or microglia cultures *in vitro* are effective strategies to explore molecular pathways involved in neuroinflammation, they cannot capture the *in vivo* interactions between neurons, astrocytes, and microglia. To better understand the effects of cellular crosstalk on neuroinflammation, new multicellular co-culture models are needed. Recently, more complex co-cultures have been developed using 2D triple co-culture (tri-culture) models of neurons, astrocytes, and microglia to study intercellular interactions and crosstalk. An example is a murine tri-culture system to study Alzheimer’s disease (AD) and recapitulate the pathophysiological characteristics lost in typical primary cultures [43]. In a tri-culture paradigm, these cells behave more physiologically than when cultivated in primary cultures: microglia exhibit less inflammation, astrocytes are less reactive, and neurons have a more mature morphology [43,67]. This 2D tri-culture model, which was found to closely mimic the *in vivo* CNS environment in a study of AD processes, should be considered for other neurological diseases such as ALS [43].

In comparison to hiPSC-derived 2D co-culture or tri-culture models, 3D organoids have enormous potential in studying disease mechanisms and neuronal development, although they require more resources than murine cultures and are methodologically more difficult. However, developments in culture technologies, biomaterials, and microfluidic devices have made it easier to move from 2D cultures to multicellular 3D model systems [21].

## 4. HiPSC-Derived Organoids

**Figure 2:**
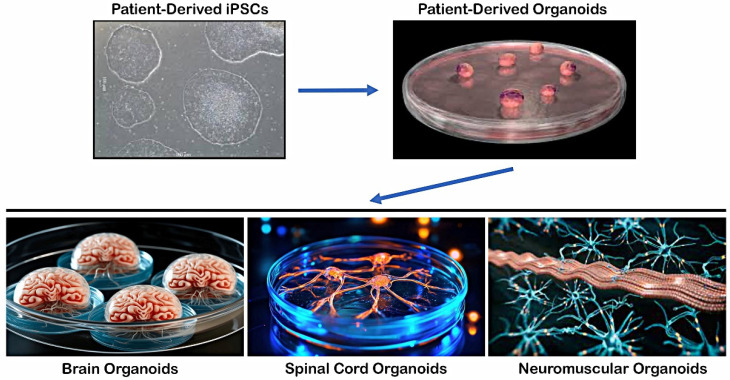
Differentiation of hiPSCs into 3D organoids and tissue-specific structures.

In contrast to previously discussed 2D models, iPSC-derived organoids present a prospective means of creating humanized 3D tissue with a variety of cell types and can provide a more accurate representation of the physiological environment [68]. Three-dimensional cell culture organoids, which can be generated from hiPSCs; multipotent adult stem cells (ASCs); or embryonic stem cells (ESCs) could create precise human disease models and provide patient-specific tissue sources for regenerative medicine. Typically, organoids are made up of several stem cells that have been cultivated with a variety of growth factors or media mixtures, causing the cells to take on specific fates that resemble the organ in question. Organoids offer a wide range of experimental applications to investigate human development and diseases, since they can be utilized to establish clinical models for tissue engineering and medication testing [69,70]. These *in vitro* culture systems can replicate certain activities of the represented organ and are distinguished by the self-organization of several organ-specific cell types into an *in vivo*-like spatial arrangement [71]. Organoids produced from patient iPSCs are more effective in expressing disease phenotypes, especially for complicated diseases in which there are no suitable disease models or when there is restricted access to presymptomatic patient samples (Figure 2).

Patient-derived iPSCs can be differentiated into multiple lineages upon exposure to specific signaling pathways and growth factors to form various tissue-specific structures, such as brain organoids resembling the cerebral cortex, hippocampus, or midbrain regions, spinal cord organoids containing anterior horn cells and neuroglia with central canal structures, and neuromuscular organoids in which anterior horn MNs with neuroglia form neuromuscular junctions on skeletal myocytes.

### 4.1. Brain Organoids

In 2013, Lancaster et al. applied a 3D culture to create the first human brain organoids that included cell lineages from the retina, choroid plexus, midbrain, and forebrain [72]. Brain organoids offer a useful model for researching neurodegenerative diseases. ALS patients’ brain organoids have been the subject of numerous studies, and the majority of these studies have focused on corticoid brain organoids [72,73,74,75].

Using the air–liquid interface (ALI) method to culture brain organoids has resulted in extended neuronal survival (e.g., >12 months), axon outgrowth with a variety of morphologies, and active neural networks, presumably because of an enhanced supply of oxygen [70]. ALI-brain organoids enable the investigation of neurodevelopmental disorders of the corpus callosum, imbalances in neural circuits, and other defects where connectivity is believed to be involved. Szebényi et al. created a human cortical organoid (CO) model that can simulate the early molecular pathology of ALS/FTD over an extended period of time [75]. COs with the *C9orf72* HRE mutation (C9 ALI-COs) were developed from iPSCs generated from individuals with ALS/FTD up to 240 days *in vitro* at the ALI. ALI-COs develop a consistent microarchitecture and mature cortical circuit-forming disease-relevant phenotypes. Although lacking microglia and vasculatures, C9 ALI-COs show abnormalities specific to neurons and astrocytes. By using a combination of single-cell RNA sequencing and biological assays, disturbances were noted in distinct transcriptional, proteostasis, and DNA repair processes in neurons and astroglia. Deep-layer neurons showed the toxic DPR species poly-GA, DNA damage, and cell death that was pharmacologically induced by enhancing proteostasis and that was inhibited by GSK2606414. GSK260414 is a selective inhibitor of the enzyme protein kinase R (PKR)-like endoplasmic reticulum kinase (PERK), which mediates the unfolded protein response (UPR) pathway that is implicated in the pathogenesis of various neurodegenerative diseases [76]. The autophagy signaling protein p62 was also elevated in astrocytes. In this model, long-term ALI-CO cultures offer an opportunity to investigate the timing and causative relationships between synapse and neuronal loss, which previously could not be examined mechanistically in the setting of human diseases [75].

### 4.2. Spinal Cord Organoids

Spinal cord organoids (SCOs) have been created from hiPSCs to study the LMN components of ALS [77]. The cellular and molecular abnormalities in ALS spinal cord can be modeled over time *in vitro* and studied by RNA-sequencing (RNA-seq), single-cell (sc) RNA-seq, and other high-throughput sequencing approaches. Creating ALS SCOs offers a unique opportunity to study LMN disease mechanisms involving both neuronal and non-neuronal cell types. In a study generating wild-type (WT) iPSC lines from healthy donor fibroblasts, Guo et al. [77] suppressed C9orf72 expression by lentivirus transfection of silencing short hairpin RNA (shRNA). Stable C9-knockdown hiPSC colonies (C9-hiPSCs) were selected and differentiated into MNs, astrocytes, and SCOs to examine gene expression of inflammation using RNA-seq (with data analysis using NCBI Gene Expression Omnibus) and qRT-PCR. Grown in a 3D culture environment, WT-iPSCs and C9-hiPSCs both retained a comparable capacity to differentiate into astrocytes, MNs, and the three germ layers to generate SCOs. In order to optimally construct mature and stable C9-hiPSC-derived SCOs, the bone morphogenetic protein pathway was blocked, and the Wnt pathway was activated; cultures were caudalized using retinoic acid and ventralized using the sonic hedgehog pathway agonist, purmorphamine. The SCOs created with lower levels of the C9orf72 protein showed characteristic cellular compositions that were similar to those in the spinal cord, including motor, sensory, and other neurons. However, the expression of inflammatory factors (e.g., IL-6 and TNF-a) in C9-SCOs was significantly higher than in astrocyte or MN 2D monolayers, indicating the involvement of numerous other nerve cell types in C9-induced neuroinflammation. Bioinformatics analyses confirmed elevated proinflammatory factors in C9-hiPSC-derived 2D cells and 3D-cultured SCOs. By using these methods, spinal neurogenesis was effectively duplicated, including the formation of a central canal, and a useful *in vitro* neural model for examining the LMN pathophysiology of C9-ALS was produced [77].

### 4.3. Neuromuscular Organoids

In the early stages of ALS, skeletal muscle and neuromuscular junction (NMJ) changes have been documented, which may be responsible for metabolic dysfunctions and in the “dying back” phenomenon of MNs [78]. The so-called “dying back” hypothesis suggests that retrograde signals contribute to the centripetal MN degeneration in ALS [78]. Skeletal muscle has gained importance in the past few years, both in the etiology and therapy of ALS [79]. Gaining insight into the molecular processes behind skeletal muscle degeneration could help in the creation of treatment strategies that maintain muscle function, halt disease progression, and enhance the quality of life for ALS patients [79]. In a recent study, hiPSC-derived neuromuscular organoids (NMOs) simulated the spinal neuromuscular characteristics of ALS patients with the *C9orf72* HRE mutation [80]. ALS NMOs replicated peripheral abnormalities in ALS, such as muscle contraction weakness, neural denervation, and the loss of Schwann cells. DPRs and RNA foci were seen in neurons and astrocytes of ALS NMOs with more prominent DPR aggregation in astrocytes. Interestingly, brain organoids derived from *C9orf72*-hiPSCs developed poly-GA aggregates in only cortical neurons; future research should explore whether this is so. The potential use of NMOs for preclinical drug testing was demonstrated after acute administration of the UPR inhibitor GSK2606414 resulted in a two-fold increase in muscle contraction and decreased autophagy and DPR aggregation [80]. Further examination of ALS NMOs generated with iPSCs from individuals with sALS or fALS due to *C9orf72*-HRE and other mutations is necessary to ensure their broad applicability to understanding pathogenic mechanisms of the LMN component of ALS.

The distinct anatomical and physiological characteristics of the human NMJ make it susceptible to pathogenic processes [72,73]. There is evidence that the pathophysiological cascade of ALS begins at the NMJ because of synaptic dysfunction and elimination occurring prior to MN loss [81]. NMJs offer the most significant pathological and functional disease readouts, and they appear to be an early and vulnerable target for ALS and spinal muscular atrophy, a genetic LMN-predominant MND [82]. Consequently, cell culture techniques facilitating NMJ development and connection to their target muscle cells will enhance the study of human MNs in health and disease [81]. Although several studies have demonstrated significant advances in the creation of skeletal muscle–spinal MN 2D and 3D co-culture procedures, modeling NMJs using hiPSCs has proven to be challenging [83,84]. Several studies demonstrated a great deal of advancement in the creation of 2D and 3D co-culture procedures. Bakooshli et al. [85] developed a 3D co-culture of healthy human muscle progenitors from a muscle biopsy mixed with human pluripotent stem cell-derived MNs that self-organized and formed healthy functional NMJs *in vitro*. However, NMJs derived from iPSCs of ALS patients have not yet been successfully produced [86,87,88,89,90]. To create and have a better understanding of NMJs’ mechanism, MNs produced from hiPSCs were co-cultured with myoblasts grown from a single control cell line in a new microfluidic technique previously [91]. The success of using *in vitro* neural networks derived from hiPSC-derived neuronal cultures and organoids to understand pathogenic mechanisms in ALS and other MNDs, as well as for preclinical drug screening, will likely depend on the ability to reproduce physiologic synapses.

In an interesting study, sensorimotor organoids containing physiologically functional NMJs were generated from five iPSC lines obtained from two healthy controls and three individuals with ALS [92]. After growing in suspension to form spheres, free-floating sphere cultures were fused, plated at a density of 46 spheres/cm^2^, and grown under adherent conditions for up to 15 weeks. Organoids containing NMJs along with skeletal muscle, MNs, astrocytes, microglia, and vasculatures, were derived and characterized using a variety of molecular, genomic, and physiological approaches [92]. Demonstrating abnormal NMJs in organoid cultures made from hiPSC lines carrying various gene mutations by physiological (contraction) and immunocytochemical approaches is significant, since NMJ loss is an early and crucial component of ALS models. Creating isogenic control pairings of such iPSC lines carrying *TARDBP*, *SOD1*, and *PFN1* mutations in this study allowed for more detailed investigations and further verified the robustness of these sensorimotor organoids. Compared to either the single parental or other lines, for example, the variation in both sphere makeup and derived cell types was dramatically reduced in the isogenic lines [92]. Human *in vitro* disease modeling of the NMJ may be useful in testing mechanistic theories of early pathogenesis in ALS, facilitating the identification and validation of targets and developing personalized preclinical therapeutic candidates. In this regard, the NMJ may already offer the first and most significant pathological and functional readouts of ALS [92,93].

In a comparable study, a 3D hydrogel-based *in vitro* ALS model was created with hiPSC-derived MNs and myoblast-derived human skeletal muscle tissue, which consistently produced axons and NMJs connecting with myotubes [81]. Human skeletal myoblasts differentiated into fully developed muscle tissue, with calcium imaging and acetylcholine-induced contraction used to verify muscle function. Two iPSC lines generated from ALS patients with *SOD1* mutations (heterozygous R115G mutation and homozygous D90A mutation) were differentiated into MNs and then co-cultured with muscle tissue. Interestingly, the 3D co-cultures containing MNs from patients or healthy controls were identical in appearance with comparable glutamate-induced muscular contraction after 14 days in a culture. This suggests functional integration of the ALS MNs and NMJs with typical morphological characteristics such as postsynaptic folding. After 21 days, however, reduced muscle contraction was observed in co-cultures made from both *SOD1* mutations; nonetheless, co-cultures were viable and functional for at least six weeks. These findings prove that MNs with *SOD1* mutations successfully adapt into the system initially and only later exhibit a pathogenic phenotype [81]. This MN–muscle co-culture model appears suitable for researching both the development and disorders of the human NMJ. However, there is still a need for a more functional, generic NMJ model applicable to a variety of conditions. Key findings in select iPSC-derived ALS model studies are shown in the Table 1.

Organoid culture techniques have advanced recently, although important challenges remain when using this multicellular approach to model neurodegenerative disorders like ALS. In addition to variability in cell composition, morphology, and maturity across preparations, the lack of the vascularization restricting provision of nutrients and oxygen, limits growth, survival, and possibly the final stages of cell maturation and differentiation. Other approaches, such as microfluidic chip (“organ-on-a-chip”) methodologies, can circumvent some of the limitations of organoids, although they are more complicated to prepare and may support less 3D analysis [94].

## 5. Discussion

Advances in hiPSC technology have unlocked new opportunities in ALS research, allowing patient-specific cells to be used for disease modeling and drug discovery. For over a century, conventional 2D cell culture techniques have contributed significantly to the *in vitro* research of various human diseases. Because such models allow for only side-by-side (single plane) contact between cells and lack 3D tissue complexity, however, they cannot accurately represent the complex nature of human cells and their interactions *in vivo*. As a result, such restrictions in the modeling process affect cell morphology, survival, proliferation, and differentiation, and consequently the mechanisms underlying human disease, including ALS [21].

Cell co-culture models allow for the study of cellular interactions with one another and the surrounding microenvironment. Besides direct contact, chemical signals within the microenvironment also play a significant role in cell–cell interactions [21]. The advent of 2D co-culture models in ALS has replicated some of the intricate connections between neuronal and neuroglial cells *in vitro*. Such studies provide proof-of-concept methods to investigate astrocyte and microglial effects on neuronal biology. They also pave the way for investigations into differing aspects of neuro-immune interactions within the framework of disease biology, using astrocytes, microglia, and neurons derived from patients suffering from various neurodevelopmental, neuropsychiatric, and neurodegenerative disorders. To fully understand the development of human MN degeneration in ALS, the roles of microglia and astrocytes must be considered. This is especially important given the non-cell-autonomous nature of ALS [95]. For example, in addition to being resident immune cells of the CNS, microglia play roles in synapse formation, the regulation of synaptic plasticity, and neural circuit maturation. Co-culturing hiPSC-derived microglia with neurons has demonstrated that microglia are phagocytically competent, express markers specific to human microglia and genes linked to neurodegenerative diseases, downregulate pathogen-response pathways, upregulate homeostatic pathways, and stimulate anti-inflammatory responses [15,45,56].

Multi-dimensional triple co-cultures of neurons with astrocytes and microglia more accurately mimic *in vivo* neuroinflammatory reactions than do conventional monocultures [43]. In 2D triple co-cultures, neurons become more complex in morphology and express higher levels of postsynaptic markers. Microglia express more anti-inflammatory markers and less pro-inflammatory markers with astrocytes, reducing the expression of pro-inflammatory markers [43]. Beyond 2D cell culture models, 3D organoid models may be the bridge to animal models by more precisely simulating *in vivo* neuronal architecture. It is likely that 3D neurodegenerative disease models are more realistic than triple co-culture 2D models, in part because of their organ-like intercellular relationships. Although techniques to reproducibly create and evaluate 3D cultures still need to be refined, they have the potential to model diseases with a higher degree of complexity and permit more intricate and *in vivo*-like communication between cells and the microenvironment. Lack of vascularization and restricted circulation to the organoid core for the delivery of nutrients and the elimination of waste products is a limitation for 3D culture models, often resulting in cell death at the center. Mixing cells of interest with mesenchymal and endothelial cells to encourage vascularization is one potential tactic to overcome this limitation [15]. Other challenges of creating such heterogeneous 3D culture models are their cost, the need for specialized equipment and optimization and consistent reproducibility from batch to batch. Nonetheless, producing more complex phenotypes is essential to recreating the most *in vivo*-like ALS disease cell culture models [15].

**Figure 3:**
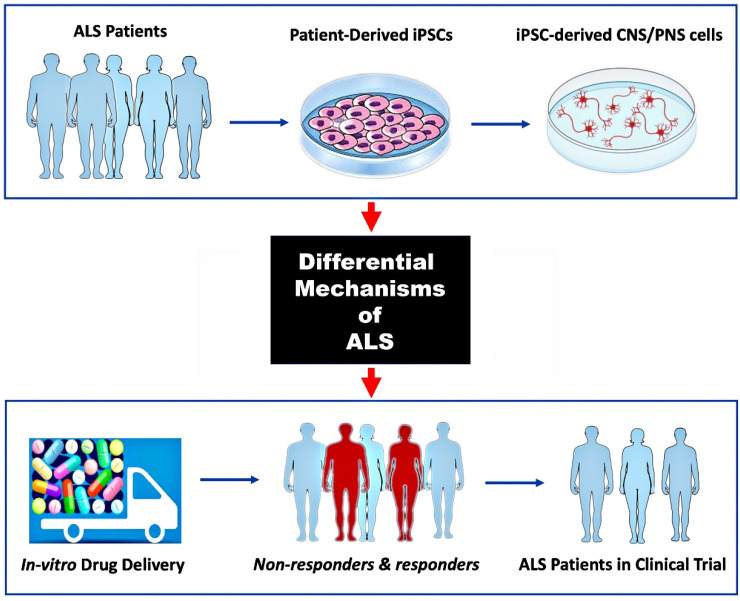
Use of hiPSC-derived ALS models for preclinical drug screening.

Our general understanding of the fundamental disease pathways of ALS is derived from years of research using both *in vivo* and *in vitro* models as well as post-mortem tissue. Important insights have been gained using these approaches, albeit with limited effect on therapeutic drug development. Because of the disease’s high mechanistic complexity, genetic heterogeneity, and translational gaps between model and patient, there is still no known cure or clinically effective treatment for ALS. Although the Food and Drug Administration (FDA) previously approved riluzole and edaravone for all forms of ALS and tofersen for SOD1-positive ALS, additional pharmacotherapies are urgently needed [96]. Drug development in neurological diseases and nervous system disorders has an estimated success rate of 8% from Phase I to FDA approval, whereas for ALS drug development, the failure rate is close to 100%. In addition, the translational potential of preclinical animal models to human clinical trial outcomes has been very poor in ALS. The hope is that many of the limitations of preclinical drug testing in animal models and the challenges of differential disease mechanisms in ALS can be overcome by using ALS patient-derived iPSCs to generate MNs and other relevant cell types to model diseases *in vitro* (Figure 3). As explained in different parts of this and other reviews [10,21,97,98,99], the development of hiPSC technology has made it possible to replicate many 2D and 3D ALS model diseases for drug development and also by using cell co-culture models to observe how cells interact with one another or with their surrounding microenvironment.

Previous availability of only animal models and the molecular heterogeneity of ALS disease pathogenesis are two of the greatest obstacles to clinical trial success. Generating MNs and other relevant CNS and/or PNS cells from ALS patient-derived iPSCs can reveal specific molecular mechanisms of disease to be targeted (top and middle panels). Screening multiple drug therapies on such 2D or 3D tissue culture models can identify patient subgroups that are more likely to respond to a particular therapy (bottom panel; non-responders in red).

This relatively recent utilization of patient-derived iPSCs to develop *in vitro* stem cell models has already advanced our molecular understanding of ALS. Because of their increasing complexity and great potential, hiPSC-derived CNS and PNS cell models represent invaluable resources to understand the basic science of and to develop novel therapies for ALS and related disorders [38]. However, several limitations in hiPSC-derived models persist, including cellular variability, lack of complexity in 2D models, and challenges in mimicking ALS-specific phenotypes [100]. One of the key challenges with hiPSC-derived ALS models is the inherent variability between different patient iPSC lines, primarily when derived from individuals with genetic diversity. Genome editing tools, such as CRISPR-Cas9 and newer base-editing technologies, offer the potential to standardize ALS models and reduce variability by precisely manipulating genetic mutations associated with ALS, such as *C9orf72* expansions, *SOD1* mutations, or TDP-43 mislocalization. Researchers can create more homogeneous disease models by introducing specific ALS-related mutations into isogenic iPSC lines, which reduces gene-related variability across individual cell lines and promotes reproducible phenotypic analysis [100].

Furthermore, traditional 2D cultures of iPSCs often fail to fully recapitulate the complexity of tissue architecture and cellular interactions seen *in vivo*. Three-dimensional culture systems, including organoids, spheroids, and microfluidic devices, offer more advanced models for ALS that can mimic the *in vivo* environment more closely. So, generating 3D MN organoids from iPSCs can create more representative disease models. These organoids can incorporate glial cells like astrocytes and microglia, which are involved in ALS pathogenesis, providing a more complete model of disease pathology. More recently, devices such as lab-on-a-chip platforms allow for the integration of multiple cell types (MNs, astrocytes, and microglia) in a dynamic 3D environment, enabling the modeling of disease progression, cellular interactions, and drug responses in real time [101,102]. Utilizing 3D bioprinting technology to create customized tissue models can improve the spatial organization of ALS disease models, enabling more precise studies of neuronal networks, axonal degeneration, and glial involvement. Additionally, Omics approaches, including genomics, transcriptomics, proteomics, and metabolomics, play a crucial role in providing a systems-level view of ALS biology and disease progression. Integrating these technologies with iPSC-derived ALS models offers several advantages for drug discovery and mechanistic insights [103].

## 6. Conclusions and Future Direction

The advent of hiPSCs to model human neurodegenerative diseases *in vitro*, including ALS, is accelerating our understanding of the molecular mechanisms underlying neurodegeneration in genetic and sporadic forms of a disease, where animal models are unavailable. HiPSC-derived single-cell-type and 2D co-culture systems have already revealed essential insights into ALS-specific cellular and molecular pathways. They are transforming the future of ALS research by personalizing disease modeling, uncovering pathogenic mechanisms, personalizing approaches to therapy, accelerating drug discovery, and opening doors to regenerative medicine. Recent developments of 3D organoid systems recapitulate a more *in vivo*-like environment of cell–cell interactions where the complexity of multiple cell types contributing to ALS causation can be closely analyzed. We are only beginning to see the potential of such *in vitro* disease models to unravel the complexities of ALS and to develop targeted therapies. As hiPSC technology advances to address the current limitations of 2D and, especially, 3D co-culture systems, it will enable more accurate modeling of human diseases, paving the way for a precision medicine approach to understanding and solving the puzzle of ALS.

## Figures and Tables

**Table 1:** Summary of findings in recent iPSC-derived ALS model studies.

Cell Type	Objective/Study	Key Findings
**iPSC-derived MNs**	sALS iPSC-derived MNs [11]	ROPI is identified as a promising drug candidate. Lipid peroxidation and resultant ferroptosis are critical factors for MN degeneration in most sALS models [11].
	hiPSC-derived MNs [27]	Sex and cell composition are significant sources of heterogeneity. Compared to controls, ALS cases have greater instances of ISL1-positive MN staining [27].
	ISL1 and LHX3 transgenes were delivered by CRISPR/Cas9 techniques in the human iPSC genome [33]	The unidirectional transition to mature MNs does not occur. Dysregulation of metabolic and regulatory pathways during development alters MN electrophysiological responses, neuronal development, cell proliferation, and Ca^2+^ signaling pathways [33].
	hiPSC-derived phMN-enriched cultures [35]	Calibrated activation of RA and SHH signaling in hiPSC-derived NPCs facilitates a cervical identity of dorsal NPCs to produce phMN-like neurons [35].
**Co-culturing hiPSC-derived MNs with Neuroglia**	Co-cultured *FUS*-ALS iPSC-derived astrocytes and MNs in a microfluidic device with skeletal myocytes [48]	FUS astrocytes are deleterious to MN neurite outgrowth, network integration, and neuromuscular junction (NMJ) formation and functionality [48].
	iPSC-derived human astrocytes co-cultured with Hb9-GFP mouse MNs [53]	Astrocytes are significantly involved in transferring mutant *C9orf72*-related DPRs to nearby MNs. The short-length DPRs used *in vitro* in this study may affect cellular functions differently from longer DPRs found *in vivo* [53].
	Co-cultured hiPSC-derived microglia–MN [61]	Unstimulated *C9orf72* mutant microglia have a dysregulated supernatant profile that does not affect the survival or activity of normal/healthy spinal MNs. *C9orf72* mutant microglia co-cultured with healthy MNs induce their apoptosis following LPS exposure [61].
**HiPSC-derived Organoids**	C9 ALI-COs developed from ALS/FTD iPSCs [72]	ALI-COs develop a consistent microarchitecture and mature cortical circuit-forming disease-relevant phenotypes. Although lacking microglia and vasculatures, C9 ALI-COs show abnormalities specific to neurons and astrocytes [72].
	C9-knockdown hiPSCs differentiated into MNs, astrocytes, and SCOs [74]	SCOs created with lower levels of the *C9orf72* protein show characteristic cellular compositions similar to those in the spinal cord and considerably increased inflammatory markers [74].
	hiPSC-derived NMOs with the *C9orf72* HRE mutation [77]	DPRs and RNA foci are seen in neurons and astrocytes. Treatment with GSK2606414 results in a doubling of muscle contraction, decreased autophagy, and DPR aggregation [77].
	Functional NMJs generated from five iPSC lines [89]	NMJs in organoid cultures show abnormal contraction and immunocytochemistry, with their early loss being a crucial component of ALS models [89].
	*SOD1* hiPSC-derived MNs co-cultured with myoblast-derived human skeletal muscle in a 3D hydrogel-based model [78]	MNs with *SOD1* mutations initially display normal morphology (e.g., postsynaptic folding) but later exhibit a pathogenic phenotype [78].

## Data Availability

No new data were created or analyzed in the preparation of this review. Data sharing is not applicable to this article.

## Abbreviations

**2**D	Two dimensional
**3**D	Three dimensional
**A**LI-COs	Air–liquid interface–cerebral organoids
**A**LS	Amyotrophic lateral sclerosis
**A**LS-FTD	Amyotrophic lateral sclerosis with frontotemporal dementia
**C**NS	Central nervous system
** C ** RISPR-Cas9	Clustered regularly interspersed short palindromic repeats
**D**PR	Dipeptide protein repeat
**E**SCs	Embryonic stem cells
**f**ALS	Familial amyotrophic lateral sclerosis
**F**US	Fused in sarcoma
**F**TD	Frontotemporal dementia
** h ** iPSCs	Human induced pluripotent stem cells
**H**RE	Hexanucleotide repeat expansion
**i**PSCs	Induced pluripotent stem cells
**L**MC	Lateral motor columns
**M**MC	Median motor columns
**M**Ns	Motor neurons
**N**MOs	Neuromuscular organoids
**N**PCs	Neuronal progenitor cells
**P**NS	Peripheral nervous system
**p**hMNs	Phrenic motor neurons
**R**AN	Repeat-associated non-AUG
**R**OPI	Ropinirole hydrochloride
**s**ALS	Sporadic amyotrophic lateral sclerosis
